# Biobased Materials from Microbial Biomass and Its Derivatives

**DOI:** 10.3390/ma13061263

**Published:** 2020-03-11

**Authors:** Celeste Cottet, Yuly A. Ramirez-Tapias, Juan F. Delgado, Orlando de la Osa, Andrés G. Salvay, Mercedes A. Peltzer

**Affiliations:** 1 Materials Development and Evaluation Laboratory (LOMCEM), Department of Science and Technology, National University of Quilmes, B1876BXD Bernal, Argentina; celestecottet@gmail.com (C.C.); yulyramirez@gmail.com (Y.A.R.-T.); juan.delgado@unq.edu.ar (J.F.D.); odelaosa@unq.edu.ar (O.d.l.O.); asalvay@unq.edu.ar (A.G.S.); 2 Scientific Research Commission (CIC), B1900 La Plata, Buenos Aires, Argentina; 3 National Scientific and Technical Research Council (CONICET), C1425FQB CABA, Buenos Aires, Argentina

**Keywords:** biobased materials, biopolymer resources, microbial biomass, yeast biomass, fungal biomass, water kefir grains, milk kefir grains, bacterial cellulose, kombucha

## Abstract

There is a strong public concern about plastic waste, which promotes the development of new biobased materials. The benefit of using microbial biomass for new developments is that it is a completely renewable source of polymers, which is not limited to climate conditions or may cause deforestation, as biopolymers come from vegetal biomass. The present review is focused on the use of microbial biomass and its derivatives as sources of biopolymers to form new materials. Yeast and fungal biomass are low-cost and abundant sources of biopolymers with high promising properties for the development of biodegradable materials, while milk and water kefir grains, composed by kefiran and dextran, respectively, produce films with very good optical and mechanical properties. The reasons for considering microbial cellulose as an attractive biobased material are the conformational structure and enhanced properties compared to plant cellulose. Kombucha tea, a probiotic fermented sparkling beverage, produces a floating membrane that has been identified as bacterial cellulose as a side stream during this fermentation. The results shown in this review demonstrated the good performance of microbial biomass to form new materials, with enhanced functional properties for different applications.

## 1. Introduction

Due to the strong public concern about plastic waste, the development of biobased polymers is gaining more and more importance in research and industry. The polymer industry relied completely on the use of petroleum since the mid-20th century. Since then, many changes appeared in the plastic industry related to the environmental impact that petroleum-based plastics may produce because of their deliberated use. Due to this, sustainable materials were developed around the 1980s [1], and new biodegradable polymers were developed and commercialized, for example poly(lactic acid) (PLA), poly(hydroxy alkanoates) (PHAs), and other polymers, in order to solve many waste problems in agriculture, marine fishery, and construction industries [2]. Twenty years later, two main problems concerning the polymer industry appeared: global warming and the reduction of fossil resources. A possible solution was found in the use of sustainable instead of fossil-based resources, for instance biomass feedstock. Biomass has been combined with chemical pathways for the production of bio-ethanol, bio-diesel, and bio-olefins [3,4]. In addition, biomass could be used as feedstock for polymer production, and these polymers are called “*biobased polymers*” [1]. This term refers to sustainable polymers (or materials) synthesized from renewable resources such as biomass instead of using fossil resources such as petroleum oil and natural gas, preferably based on biological and biochemical processes. They are characterized by being carbon neutral or carbon offsetting, by which atmospheric CO_2_ concentration does not change even after incineration [5]; in addition, biobased materials could be produced from residues [6]. Biobased polymers include three classes of polymers: *biopolymers*, *synthetic polymers*, and *bioengineering polymers*. “*Biopolymers*” are naturally occurring polymers formed by plants, animals, and microorganisms. In this group naturally occurring and chemically modified polymers are included, such as cellulose, cellulose acetate, starches, modified starches, chitin, gelatin, vegetal proteins, β-glucan, dextrane, and kefiran. Biopolymers are directly used as obtained from their sources and are biodegradable; they are also referred to as natural polymers. Their industrial use depends on the source of the materials: plants, microorganisms, and marine algae. Alginate, cellulose, carrageenan, and starch are commercially used polysaccharides with a wide range of applications mainly in the food and pharmaceutical industry, but there are other applications [7]. The main drawback with these polymers is the diverse quality and lack of assured supply. Regarding the group of biobased “*synthetic polymers*”, we found poly(lactic acid) (PLA), poly(butylenes succinate) (PBS), bio-polyolefins (bio-PP, bio-PE), and bio-poly(ethylene terephtalic acid) (bio-PET). In this group, PLA and PBS are biodegradable/compostable polymers, and PLA is the most promising biodegradable polymer for different applications, such as food packaging [8,9], automotive applications [10], and biomedical applications [11,12], among others. Bio-PP, bio-PE, and bio-PET are not biodegradable materials, and the only contribution for reducing environmental impact comes from reducing the carbon footprint. The origin of a polymer does not determine its biodegradability; this condition depends on the chemical structure of the polymer [13]. Then “*bio-engineering polymers*” refers to those polymers biosynthesized by microorganisms, and this is the case for the poly(hydroxyl alkanoates) (PHAs). These materials are natural polyester polymers synthesized by bacteria, and they are 100% biodegradable [14].

Regarding the concept of environmentally friendly materials, is not possible to say if a material is good or harmful for the environment. It is necessary to evaluate, in each case, the interaction with the environment, the conditions used during the production of the material, the additives which are present in the material formulation, the use that consumers give to the material, and finally, the disposal of the material after their shelf-life. In the case of biobased materials there is an advantage from the point of view of using renewable resources and plants [15]. Besides these advantages, it is important to consider the conditions of harvesting of the species used to produce plastics [16], since there could be a negative impact to the environment due to the depletion of soil nutrients, deforestation, use of fertilizers, and pesticides [15]. There is also the controversy of using the farmland to produce plastics or biofuel instead of food [17], in addition to the increase in the price of basic cereals such as corn. Alternatively to the vegetable origin biopolymers, microorganisms synthesize a wide variety of exopolysaccharides (EPS), composed mainly of glucose, mannose, fructose, and galactose and also uronic acid and other non-carbohydrates compounds, such as acetate, pyruvate, succinate, and phosphate. Examples of EPS are “bacterial cellulose”, from *Acetobacter sp*, *Gluconoacetobacter xylinum*, and *G. hansenii*, among other bacteria [18,19]; “kefiran and dextran” from kefir microflora [20,21]; “pullulan” from *Aereobasidium pullullans* [22]; “levan” from *Bacillus polymixa* [23], *Leuconostoc mesenteroides* and *Lactobacillus reuteri* [24], among other microorganisms [7]; and “gellan” from *Sphingomonas paucimobilis* [25]. The EPS production depends strongly on physical and chemical factors. Physical factors are temperature, agitation, and aeration; and among chemical factors are media composition, source, carbon and nitrogen concentration, pH, and dissolved oxygen, which affect polysaccharide production. Indeed, the cells of microorganisms (microbial biomass), for example of yeast and filamentous fungi biomass, are composed mainly of biopolymers such as proteins and polysaccharides (β-glucan) [26,27,28]. Yeast biomass, in addition to its conventional applications in fermentation processes, could be used in alternative applications such as encapsulation of compounds [29], films and coatings [30,31], among other applications. Filamentous fungi biomass is mainly used for human consumption, but fungal enzymes and bioactive compounds are widely used in the food industry and in veterinary and human medicine [32,33,34]. Not only the metabolic products of filamentous fungi are interesting for research and industry, but also the mycelial structure is promising for new application areas. For example, for the fabrication of structures that contain vegetal fiber as a filler which is glued by fungal mycelium [35,36]. In addition, development of sustainable products by using fungi biomass was described for the textile, packaging, and automotive industries [28]. 

The benefit of using microbial biopolymers for industrial production is that it is not limited by crop failure, climate conditions, or marine pollution. Some of these biopolymers could be more expensive than the plant-based polymers; however, research on new methods and the use of low-cost culture media, such as alternative carbon and nitrogen sources, may reduce the production of more expensive EPS [7]. Yeast and fungal biomass are low-cost and abundant sources of biopolymers, since they could be a residue from some industrial processes such as the brewing industry [31,37] or other biotechnological processes [38] where the biomass (cells or mycelia) is discarded after obtaining the final product. 

The present review is mainly focused on biobased materials using microbial biomass and its derivatives, focusing the attention on yeast and fungal biomass, water and milk kefir grains, and bacterial cellulose produced by fermentation of specific bacteria and from kombucha teas. Throughout this review, the authors show the promising characteristics of these types of biomass for the development of sustainable materials for different applications with the aim of exploiting new sources of *biobased* materials. 

## 2. Fungal Biomass as Source of Materials

### 2.1. Yeast Biomass as Source of Biobased Materials

Yeasts are eukaryotic unicellular organisms that are included in a large group called “fungi”, which also includes molds and mushrooms. They are facultative organisms which can be developed either in absence or presence of oxygen. Yeasts are well known for their fermentative properties [39], but contrary to popular belief, approximately half of the species are not able to do so. However, many of these yeasts have become a versatile tool in biotechnology [40]. Humans have used yeast for thousands of years for the production of fermented foods, such as bread, beer, and wine. At the beginning, the first uses of yeasts were instinctive but with developments in science and technology these microorganisms became the focus of several investigations for new applications [41]. The first microscopic observation of the yeast cell was reported by Antoine van Leeuwenhoek in 1680. Many years later, the contributions of Louis Pasteur in 1857 on yeast metabolism were the key to understanding the fermentation process. Twenty years later, the first pure cultures were obtained, better known as starters, to produce beer and wine by Hansen and Müller-Thurgau [40], respectively. In the past century, great advances have been made in genetic engineering to convert yeast cells into small factories because they have the ability to convert raw materials into valuable compounds that can be used for example as food additives. In this way, the demand for yeast biomass increased, favoring the production not only for food and beverages but for a wide range of products. Nowadays, yeasts are used mainly in the food industry, but there are more and more different roles that they play in other fields. 

Regarding environmental issues, yeasts can be used for bioremediation and as heavy metals removers from waste waters; in agriculture, they are used as biocontrol agents; in pharmaceutical applications, for the production of several chemicals, and for biofuel production, among others [40,42,43]. A very recent and innovative application is the formation of films and coatings in the materials industry, mainly for food packaging [30]. 

### 2.2. Yeast Cell Structure

**Figure 1:**
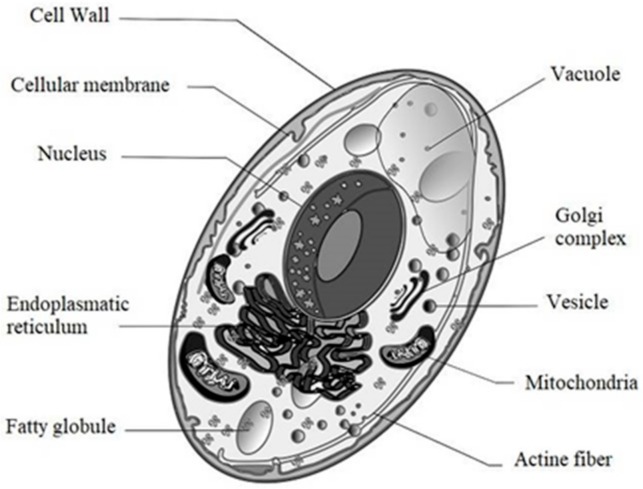
*S. cerevisiae* cell and organelles description.

Components of yeast cell have the main role in the production of biodegradable materials, in particular, proteins from cytoplasm and β-glucans from the cell wall. Yeast cell consists of an outer cover called a cell wall—very permeable but resistant to mechanical stress—composed mostly of polysaccharides (Figure 1). The major polysaccharides are β-glucans, which are polymers of glucose with β(1-3) and β(1-6) bonds. β-glucans solubility in acid/alkaline media is variable; the difference between solubility and insolubility in alkaline medium is that the former involves linear polymers with β(1-3) bonds, and the insoluble ones are highly branched polymers with β(1-6) bonds [44]. Highly branched β-glucan (1-6) bonds have a polymerization degree of 24 and an average mass of 150 kDa, while β(1-3) glucans have a polymerization degree of 240 and a mass 1500 kDa average. 

**Figure 2:**
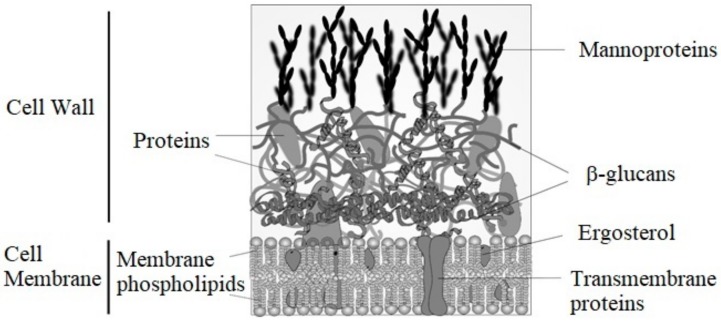
Membrane and cell wall from *S. cerevisiae.*

Hierarchically, β-glucans are arranged in the cell wall as follows: the linear ones, β(1-3)-glucans, are located in helix mode in the inner part of the wall (like a spring), able to absorb the expansions and contractions due to dehydration and rehydration (Figure 2). The β(1-6)-glucans, highly branched and generally associated with cell wall proteins, are located in the outer parts of the wall. Proteins could also be bonded directly to β(1-3)-glucans through a bond known as ASL (alkali sensitive bond). Alkali soluble β-glucans are capable of forming gels [45].

In addition to β-glucans, there are glycosylated proteins with mannose polymers, called mannoproteins, which correspond to 25% of the cell wall mass. There are at least 20 glycoproteins, and beyond the general functions of the wall, they fulfil various specific functions for the cell. Mannoproteins are located on the outside portion of the wall and are extended all through it. Mannoproteins have a structural role and consist of approximately 90% polysaccharide and 10% protein, while mannose units are also found in the periplasmic enzymes, with higher protein content. There is a fraction of mannoproteins called α, which is able to get good emulsifying properties if there is a reduction in the polysaccharide content with respect to the original, since the polysaccharide fraction presents poor properties for stabilizing emulsions [44]. Various technological applications of mannoproteins have been postulated in the production of wine, for example, adsorption of Ocratoxin A, stimulation of the growth of malolactic bacteria, inhibition of crystallization of tartrate, and flocculation of yeasts, among others [46].

The function of the cell wall is to protect the cell against external stress agents, as well as maintain the internal turgidity. Klis et al. also pointed out two additional functions of the cell wall; the maintenance of the cell shape and the necessary scaffolding for proteins [47]. The proteins related to the cell wall limit its permeability to macromolecules, closing it to an exclusion size of 740 Dalton; in addition, they protect the cell from the attack of external enzymes, prevent the loss of precursors in the construction of the wall, and contribute to the water retention with the phosphate groups of carbohydrates in glycosylated proteins.

Between the cell wall and the phospholipid membrane (Figure 2) there is a space known as periplasmic space, with the presence of enzymes that are useful to the microorganism. These enzymes are capable of degrading complex substances into smaller molecules that can pass through the membrane and nurture the cell. The plasmatic membrane is a phospholipid bilayer with protein complexes that facilitate the entry or egress of substances. It has a thickness of 7.5 nm, and the inner layer is composed of phospholipids such as phosphatidylinositol, phosphatidylserine, and phosphatidylethanolamine, while the outer layer is more enriched in phosphatidylcholine. The cytoplasm and cell wall membrane lipids are 7% of the dry matter in yeasts [48]. Unlike animal cells, where cholesterol is common, ergosterol and a small amount of zymosterol are mainly present in yeasts [49].

The cell membrane represents a minor fraction of the total cell and the primary functions of the plasma membrane are the physical protection of the cell, the control of osmotic stability, and the regulation of substance transport. It also serves as an anchor place for cytokinesis, the control of the synthesis and repair of the cell wall, and the localization of components of the transduction signal pathways. Inside the yeast cell, the organelles are located, where the functions of the cell are specialized. The internal pH is slightly acidic (5.2), and there are soluble compounds of low and medium molecular weight, such as proteins, glycogen, and other soluble macromolecules. Macromolecular entities such as ribosomes, proteasomes, and lipid particles are suspended in the cytoplasm. In the cytosol, there are several enzymatic complexes, glycolytic enzymes, enzymes for protein synthesis, and structure maintenance.

The endoplasmic reticulum and the Golgi apparatus are internal structures with the highest protein density in the cytoplasm. The endoplasmic reticulum is the key organelle where the processes that control the stability, modification, and transport of proteins are performed. It is an extended system of branched tubes covered by a phospholipid bilayer. The endoplasmic reticulum represents 10% of the cell volume. All transport proteins of the plasma membrane and the different organelles are produced in the endoplasmic reticulum. Before being transported, an assisted folding system controls the correct conformation of the proteins before migrating to the Golgi apparatus.

The Golgi apparatus consists of a series of “folded bags”, the net conformed by the *cis* bags are the entrance to the apparatus, and the *trans* bags represent the exit of it. In the Golgi apparatus, proteins, and polysaccharides are processed, with reactions such as the formation of polysaccharide complexes *N*-acetylglycosylated proteins, oligosaccharide phosphorylation, protoglycan synthesis, and lipid modification, among other reactions. There is also a reverse transport system from the Golgi apparatus to the endoplasmic reticulum. A quality control system returns proteins that were not correctly folded before being released for use. According to the isoelectric point database [50], which calculates the isoelectric points of the sequenced proteins of different microorganisms, it was observed that the distribution of isoelectric points of *S. cerevisiae* proteins presented a bimodal characteristic centered at a pH from 4 to 6 and from 8 to10.

### 2.3. Use of Saccharomyces Cerevisiae Cells as Film Forming Material

Nowadays, *S. cerevisiae* was and still is the most widely exploited yeast species in industry. Cells of this yeast are a natural, low-cost food ingredient recognized as food grade in human nutrition, with high availability to produce biobased films with inherent biodegradability. Commonly, these single celled organisms have been used mainly by the brewing, wine making, distilling, and baking industries because of their production of ethanol and carbon dioxide by fermentation of sugars. In recent years, significant interest has emerged for other promising applications. Yeast biomass contains proteins and polysaccharides—about half of its dry weight—which could be isolated giving added value to yeast production [48]. These biopolymers extracted directly from biomass are gaining more attention in the science community for the development of biodegradable materials for food packaging. 

Brewer’s spent yeast biomass has a potential to be valued, besides the bioactive compounds which are present inside the cell; yeast biomass is an important source of biopolymers to prepare biodegradable films. These advances were achieved in response to the new trend and consumer demands for natural products that create less environmental contamination [48]. There are few investigations related to the use of the integral biomass to prepare biodegradable materials. Delgado et al. prepared casted films based on integral yeast biomass [30,48]. In their work, the authors demonstrated that it was necessary to make a dispersion of yeast biomass (10% wt dry mass) from which the film will be formed. In order to obtain the yeast components able to perform a matrix which will develop the film, it is necessary to break the cell and release the cytoplasmic content by some method for cell disruption. In this way, proteins and polysaccharides are free to interact and form the film network. Usually, high-pressure homogenization breaks yeast cells releasing the intracellular material to the surrounding media. In this study, different pressures in combination with a heat treatment were applied. Pressures higher than 60 MPa were the more effective, and 125 MPa was the most accurate [48]. In addition, the treatments must be carried out in a certain order to obtain the dispersions that will form the film with the best properties, the most successful being a combination of homogenization (125 MPa), followed by a heat treatment, and then a second homogenization (125 MPa). The first homogenization disrupted the cell, then the heat treatment was applied to denaturalize enzymes and proteins, and finally a second homogenization was applied to eliminate possible aggregates that have formed during the heat treatment. Then the solvent (water in this case) must be removed, favoring the interaction between the polymeric chains and the formation of the film matrix [26]. In the investigations about yeast films, glycerol was added as a plasticizer to the already treated yeast dispersion to produce casted films [30]. Yeast films were fully characterized in their visual, thermal, infrared spectroscopy (FTIR), mechanical, and hydration properties. Plasticizers improved film integrity, flexibility, and mechanical properties because they tend to increase the molecular space between polymer chains. Thermogravimetric analysis (TGA) demonstrated that there were different zones of degradation, where the first degradation zone, from 30 to 140 °C, was attributed to water evaporation and low-molecular-weight molecule degradation. The second zone, from 140 to 225 °C, implies the beginning of the partial degradation of the protein, as proven with FTIR assays. Similar results were obtained by Guerrero et al. in films made from soy protein isolates, and they reported substantial degradation of the material above 180 °C [51]. Then, the third degradation zone extended from 224 °C to 360 °C, with the maximum degradation temperature (T_peak_) of nearly 309 °C attributed to the pyrolysis of β-glucans and to the massive decomposition of proteins and other organic compounds [52,53,54]. Finally, above 360 °C starts the last zone, which presents two more events which complete the thermal degradation of samples. When glycerol content was increased, the glass transition temperature was shifted to lower values. Maximum elongation at break was around 12%. Young’s modulus (YM) decreased from 88 to 9 MPa with the addition of plasticizer. The values obtained for YM and the maximum strength were similar to those reported in films based on soy protein isolate with significantly higher amounts of glycerol [51]. In contrast, these films of soy protein had a better performance regarding the deformation at break. The water uptake of yeast films rose from 0.49 to 0.79 g H_2_O/g of dry matter with plasticization. The characterization of yeast biodegradable films provided evidence of their properties for a potential future use in the packaging industry.

These materials should provide a good protection with good barrier properties against hydration or dehydration of the packaged food [55]. An exhaustive study of the hydration and water transfer properties on yeast films, in addition to the effect of plasticizer and film thickness, was performed. The water solubility in the film matrix and the diffusion coefficient were calculated with hydration kinetics experiments. Results demonstrated that when the glycerol content was increased, the solubility in water increased while diffusivity remained constant. Moreover, when producing thicker films, the solubility in water decreased but the diffusion coefficient increased. The relevance of this study was revealed by the increase of permeability with the plasticizer content due to the increase in solubility, while the effect of the thickness increasing permeability was dominated by diffusion [55].

### 2.4. Use of Fungal β-Glucans as Film Forming Materials

The production of yeast extract is obtained after mechanical or enzymatic hydrolysis of yeast cells. In this process, the intracellular content is isolated and used as a food additive, flavor enhancement, or for culture media, but the broken yeast cell wall is discarded as waste or used for animal feeding. The yeast cell wall is rich in polysaccharides and proteins, as commented before; hence, it also has film forming abilities. The yeast cell wall is mainly composed of β-glucans and mannoproteins which represent 30–40% of the cell, having distinct structural and physiological functions in the yeast organism [26,56]. β-glucans build the skeleton which defines cell wall stability and cell morphology; on the other side, mannoproteins constitute an amorphous matrix and cell surface fibrous material [56]. β-glucans are polysaccharides naturally present in the cell wall of various living organisms such as bacteria, yeast, fungus, mushrooms, and plants. Their properties are related to their molecular weight, chemical structure, and rheological characteristics, which may vary according to the source of β-glucans [57]. Depending on the source, β-glucans have different conformations, where those from fungi are branched, composed of a backbone chain of residues of β(1,3)-D-glucose to which side groups of β(1,6)-D-glucose are attached. Those branches give attractive properties to the molecule, such as bioactivity. Those branches from yeast cell wall present a triple helix conformation, a distinctive feature of the polysaccharide family [26]. 

Novák et al. were the first to obtain films from isolated β-glucan suspensions from baker’s yeast using glycerol as a plasticizer [52]. The films that were obtained presented a granule and fibrous microstructure and were insoluble in water, compact and non-porous, with no pronounced crystallinity. After one year of shelf storage, some structural changes in the films surface were observed, which may be due to the reaction of surface macromolecules with the ambient atmosphere.

Residual yeast cell wall (YCW) from yeast extract production was also used without any purification for films development [31]. In this study, residual YCW, rich in β-glucans, was used as the base matrix, and different concentrations of glycerol were tested to develop biodegradable films. The results of this research were that homogeneous and yellow-brownish films were easily obtained after a thermal treatment of the cell wall and the addition of NaOH to increase the pH of the media. Total soluble matter demonstrated that glycerol enhanced the solubility of films, but the plasticizer was retained in the polymer matrix. FTIR analyses confirmed the presence of proteins in discarded yeast cell wall, corresponding to the mannoproteins linked to glucans [26]. Scanning electron microscopy (SEM) photographs of the YCW films showed that the shape of the cell wall was maintained after film formation. Finally, 15 wt% of glycerol seemed to be enough to improve the mechanical properties, and a linear increment of water vapor permeability with glycerol concentration was produced by the increase in water solubility in the film. 

### 2.5. Yeast Cells as an Ideal Carrier for Encapsulated Compounds

Encapsulation is a process to entrap one compound within another one. The compound that is encapsulated may be defined as the core material, and the compound that encapsulates may be defined as the shell or coating. Another property attributed to yeast cell is its attractiveness and potential as biological vehicles for the encapsulation of different molecules, exploited in many industrial sectors [58]. There are different microencapsulation technologies such as spray-drying, freeze-drying, fluidized bed coating, extrusion, emulsification, coacervation, and electrostatic methods [59]. It is a process that takes place in the food, perfumery, agrochemical, chemical, and pharmaceutical industries, because one of the advantages it possesses is that the encapsulated compound can be released in different systems in a controlled way [60]. The stability and release properties of microcapsules depend on the wall material composition [59]. *S. cerevisiae* is again a key organism, which becomes an ideal carrier of active compounds because of their food grade and cost-effective characteristics. For yeast cells as microcapsules, the methodologies mostly used were plasmolysis of the cell, in order to eliminate cytoplasmic material, and spray-drying and freeze-drying [58]. The molecules to be encapsulated can be both hydrophobic and hydrophilic due to their behavior as a liposome [31,58,60]. One of the applications is to encapsulate volatile molecules as flavors to guarantee permanence during the industrial process or probiotics in order to optimize their viability within a thermostable molecule [61]. There are studies which show that yeast cells are stable up to temperatures close to 250 °C, which makes them great candidates to encapsulate and protect bioactive compounds [59].

### 2.6. Mycelium Biobased Materials

Another strategy to develop biobased materials from alternative sources than vegetable-derived ones is the development of new materials based on fungal biomass, in particular the mycelium. The advantage of these materials is that their properties are controlled and tunable during the growth of the microorganism, and there is no need for any extraction of biopolymers or expensive processing technology. Fungal mycelium is the largest living organism on earth [62]. It grows due to its symbiotic relationship with the materials that feed it, forming entangled networks of branching fibers [63]. Haneef et al. produced films based on mycelia of two edible and medicinal fungal species, *Ganoderma lucidum* and *Pleurotus ostreatus*, grown in different substrates and compared their properties with other biobased polymers produced by microorganism, such as bacterial cellulose and PHB [64]. In that study, the growth of the mycelium materials was done by feeding them with two natural polymeric substrates, pure amorphous cellulose and a mixture of cellulose and potato dextrose broth (PDB). The nutrients used were polysaccharide-based medium; however, the one that contained PDB was more easily absorbed by the mycelium due to its higher concentration of simple sugars. The mycelium films were heat treated at the end of their growing period in order to stop the growing of the fungus and to obtain the final fibrous membranes. The physicochemical properties of the self-grown films were defined by the physiological characteristic of the two species, and most importantly by the different feeding substrates. Since then, the authors observed that the addition of PDB in the growing media affected the secondary structure of proteins of *G. lucidum* and increased the relative concentration of lipids on films based on that microorganism, while increasing the percentage of proteins in *P. ostreatus*. In addition, there was a reduction of the relative presence of chitin in both species. These modifications had a big influence on the final mechanical properties of the mycelium-based films. When the feeding substrates contained PDB, the self-grown fibrous materials showed lower YM values and increased elongation at break. This substrate makes the mycelium less rigid and more ductile, in comparison with the cellulose-fed materials. In addition, PDB mycelia presented high degradation temperatures and more hydrophobic properties than the cellulose-fed one. The study demonstrated that mycelium-developed materials are naturally multicomponent materials that can be tuned by modifying their nutrient substrates, representing a new way of producing functional materials in large amounts with low costs. 

Filamentous fungi are also an important source of β-glucan and could be added to this technology as a film-forming material. Martinez et al. proposed the initial methodology to develop multicomponent films based on biomass of the Ascomycete *Paecilomyces variotti*, in addition to the study of the effect of the sterilization on the filmogenic dispersions [65]. Aqueous dispersions (3% wt dry mass) were prepared from fungal biomass obtained in yeast extract supplemented broth (YES), during 7 days at 25 °C, the biomass (pellets) was filtered from the broth and subjected to ultrasound homogenization (US): 15 min and 80 W. Previous to the US, the biomass was sterilized (121 °C, 15 min) in two different ways. One way was to prepare the 3% wt dispersion, sterilize it, and the US treatment was applied (called NF sample); the other way was to sterilize a certain amount of biomass in water (50 g/250 mL), then filtrate the biomass, prepare the 3% wt dry mass dispersion, and then the US treatment (called F sample). Glycerol was added as a plasticizer at 25% wt dry mass, and non-plasticized systems were also prepared. Films were obtained by the casting method (35 °C, 50% RH). Thermal and mechanical properties, water vapor permeability (WVP), and browning characteristics were analyzed. Films presented a multiple-step thermal degradation profile, determined by thermogravimetry, with a maximum degradation at 260 °C corresponding to degradation of β-glucans. However, when the samples were plasticized, an additional degradation process was observed at a lower temperature, which differed between the samples F and NF, possibly corresponding to the degradation of the glycerol and its interaction with the biomolecules which are present in the material. The mechanical properties showed that both tensile strength (TS) and Young’s Modulus (YM) decreased with the addition of glycerol as expected, and those F samples had a higher TS and YM values (5 ± 2 and 80 ± 25 MPa, respectively) than those of the NF samples (1.9 ± 1.1 and 13 ± 9 MPa) [65]. There were no differences in the color of the films with the different treatments. Regarding WVP, in those samples without plasticization, no changes were observed between the treatments. However, in the plasticized samples, the NF samples have a significantly higher WVP value than the F samples. The results showed that in the NF samples, low molecular weight compounds may be present, which are more hydratable, and this may affect both the permeability and the mechanical properties of the material. 

In the end, yeasts and fungal mycelia are key organisms, which are commercially available, low cost, and important in human nutrition. With time and thanks to research, fungal biomass has become a versatile tool for different industries, with innumerable and diverse applications.

### 2.7. Applications of Fungal Biomass

Recent applications and patent developments in the field of material science demonstrated the potential of fungal biomass to be used in several technologies, which are detailed in Table 1.

## 3. Kefir Grains as Source of Materials

### 3.1. Milk Kefir Grains and Water Kefir Grains

**Figure 3:**
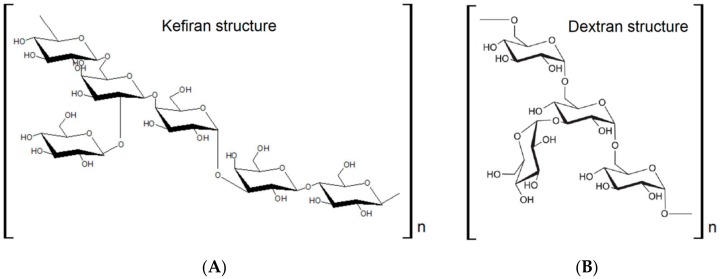
Chemical structure of kefiran (**A**) and dextran (**B**) polysaccharides.

Kefir commonly refers to a beverage for which fermentation of milk has been induced by resilient and yellowish-white granules varying in diameter from 3 to 35 mm, named ‘kefir grains’ [70]. These grains, supposedly found in the Caucasus [71], contain lactic acid bacteria (LAB), acetic acid bacteria (AAB), and various yeasts combined with casein and complex sugars in a polysaccharide matrix [72]. In kefir grains, the main polysaccharide is kefiran (Figure 3A), which is a water-soluble branched gluco-galactan heteropolysaccharide containing approximately equal amounts of glucose and galactose and is mainly produced by *Lactobacillus kefiranofaciens* [70,73]. Kefiran has been reported to possess several beneficial properties for human health, such as antioxidant, antitumor, and anti-inflammatory properties [74]. One feature of kefir grains that differs from other fermented milk products is that kefir can be recovered after fermentation with a slight increase in grain biomass [73].

On the other hand, there is another sour, alcoholic, and fruity beverage, made from sugar and water with figs and lemon added, whose fermentation is induced by a different type of kefir grains which are transparent, and mucilaginous but less resilient [71]. These grains have been given various names such as Gingerbeer plants, California bees, Tibis grains, or Tibi-complex [75]. The Tibis grains are known to originate from the Mexican cactus Opuntia where they were taken off the leaves [75]. However, uncertainty remains about the origin of the other grains. The transparent grains are commonly called sugar kefir or water kefir grains to differentiate them from the grains fermenting milk [71]. These water kefir grains consist of an extracellular polysaccharide and contain the microorganisms responsible for the water kefir fermentation [76]. The polysaccharide matrix of water kefir grains is described to contain dextran (Figure 3B), which is a water-soluble glucose polymer, mainly composed of linear α-1,6-linked with a low percentage of α-1,3-linked side chains [77]. To date, it is known that the microbial species diversity of water kefir consists of a stable consortium of mainly LAB, AAB, and, yeasts [78]. This microbiota makes water kefir a valuable source of bacterial strains with interesting physiological properties [79]. 

Several studies have reported the use of polysaccharides from different sources to prepare films and coatings with different properties and have indicated that these carbohydrates are promising materials [80]. Less attention has been paid to microbial EPS, even though these materials can form gels and viscous solutions at low concentrations [81]. Nevertheless, in recent years, a significant emphasis has been placed on the research of materials obtained from microbial EPS [20,82,83]. For commercial applications, EPS must be produced at high levels in low cost media, and the isolation procedure must be easy with high yields [84]. Taking into account these considerations, EPS from kefir grains might be an affordable alternative [82].

### 3.2. Milk Kefir Grains as Source of Biobased Materials: Kefiran-Based Materials

Kefiran can be easily isolated and purified by a simple method that consist in treating kefir grains in boiling water, then centrifuging and finally precipitating the polysaccharide in the supernatant by addition of cold ethanol [82,84]. Research works have demonstrated the capacity of kefiran from milk kefir grains to form films [82,83]. Piermaria et al. have obtained films by casting from solutions in water at a concentration of 1% wt kefiran, while Ghasemlou et al. obtained films from solutions at 2% wt kefiran [82,83]. All film-forming solutions exhibited a pseudoplastic behavior, and glycerol addition did not modify the solution rheological properties [81,82]. 

Kefiran films presented a yellowish color [83] and had comparable transparency with other polyol-plasticized films [82]. Moreover, the obtained transparency values were within the range of those of some commonly used synthetic films such as low-density polyethylene [82]. SEM photographs of films showed smooth and uniform surfaces without pores or cracks exhibiting plasticized films with more compact structures than those of unplasticized ones [82]. X-ray diffraction patterns of kefiran films confirmed an amorphous-crystalline structure without the sharp peaks associated with crystalline structures [81].

The solubility in water of the kefiran films was measured from immersion assays in 50 mL of distilled water with periodic stirring for six hours at 25 °C [83]. Unplasticized kefiran films exhibited a solubility of 21% while solubility of films with 35% glycerol was 29%. In comparison with other polysaccharide-based films, kefiran films have a higher contact angle with water, suggesting a higher surface hydrophobicity. This means that these films were less readily wetted [83].

These films exhibited water vapor permeability comparable with other biopolymeric materials obtained from more traditional sources such as starch, chitosan, methylcellulose, and sodium caseinate [82,83]. Some reports informed that the addition of 25% wt dry mass of glycerol allowed decreasing water vapor permeability [82]. These authors attributed this anomalous behavior to the development of a more compact structure in plasticized films. On the other hand, some authors observed that water vapor permeability of films increased with the plasticizer content as expected [83]. These divergences could be due to the differences in film-forming solution formulations and film-making procedures [83].

Kefiran films without glycerol were brittle and rigid since they showed high Young’s Modulus and tensile strength values and low deformation at break [82]. Glycerol addition of 25% wt led to high elongation values of 130 ± 15% [85], allowing flexibilities comparable to those of synthetic materials [82,83,84,85]. According to infrared spectroscopy studies, when plasticizer, especially glycerol, was included in kefiran films, these plasticizer molecules may have remained within the kefiran polymer, disrupting its structure [81,85]. The possible conformational changes in kefiran structure could be the reason of producing extremely flexible films when glycerol is included in the matrix. On the other hand, increased glycerol concentration caused a decrease in glass transition temperature (*T*_g_) from −14 °C for unplasticized kefiran films to −21 °C for films with 35% wt glycerol [83]. The reason is that plasticizers dissolved in the polymer, separating chains from each other, facilitating chain movement increasing flexibility and favoring a rubbery state [86].

Kefiran films with more than 35% wt glycerol were flexible but sticky [87]. Stickiness of glycerol-plasticized films might have resulted from the phase separation and diffusion of glycerol to the surface of the film. Sorbitol-plasticized films with more than 35% wt sorbitol concentration developed white spots on the film surface [87]. The white residue might be due to an excess amount of sorbitol. A similar situation was observed by Jangchud and Jangchud (1999), who explained that this occurred when the plasticizer content was more than its compatibility limits [88]. At high plasticizer concentrations, phase separation occurred, resulting in a sticky surface on the films plasticized with glycerol and crystallization in the films plasticized with sorbitol [87].

Kefiran films containing probiotic microorganisms have also been developed [89]. It is noteworthy that the microorganisms survived in kefiran films and had the same or improved resistance to gastrointestinal conditions. Thereby, kefiran matrix with health-promoting properties results appropriate for probiotic administration to be used for the prevention of gastrointestinal disorders [89].

### 3.3. Composite Materials Made with Kefiran

Furthermore, composite films based on kefiran and oleic acid, were prepared using emulsification improving their water vapor barrier properties by 33% [90]. Films prepared by blending kefiran with corn starch were also studied. It was observed that these two film-forming components were compatible, and a good interaction existed between them. It was observed that when starch was added to kefiran, the flexibility of the composite film was improved without reducing maximum tensile strength [91]. Nanoparticles of ZnO were successfully incorporated to kefiran-starch films to obtain materials with UV-protective properties [92].

Moreover, kefiran-whey protein isolate nanocomposite films were produced using montmorillonite and nano-TiO_2_ as nanoparticles [93]. Incorporation of montmorillonite into the polymeric matrix by improving the physical and mechanical properties provides a compact structure that can significantly decrease the water vapor permeability of films [93]. TiO_2_ as a metallic nanoparticle, after addition to the polymeric matrix, changed the color attributes and increased the transparency of nanocomposites films [93].

These studies show that kefiran can integrate with other biopolymers, biomolecules, and nanoparticles and interact positively to form new materials with enhanced functional properties for food packaging applications.

### 3.4. Water Kefir Grains as Source of Biobased Materials: Dextran Based Materials

Although films based on kefiran demonstrated to have interesting properties and significant elasticity, the procedure to obtain films implies the isolation and purification of the polysaccharide from milk kefir grains. The traditional approach to develop new biodegradable materials has been the purification of interesting biopolymer from their original biomass, and physical or chemical modifications to enhance their capability to form films. Recently, a new approach that contributes to a more efficient process with less waste was applied to obtain a novel material using the entire biomass of the water kefir grains [20]. The whole biomass was dispersed in water at 3% wt dry mass and submitted to physical treatments of ultrasonic homogenization separated by a thermal treatment at 90 °C. Finally, water kefir films were obtained by casting procedure of the treated water kefir dispersion [20]. Films obtained from whole biomass of the water kefir grains exhibited a great continuity and homogeneity without cracks and presented a remarkable transparency that was not observed in films made from purified kefiran [20]. Infrared spectroscopy and thermal degradation studies revealed that the material was basically constituted of the polysaccharide dextran. No differences were found in the visual appearance for films plasticized with glycerol. On the other hand, the gradual addition of plasticizer significantly increased the flexibility of the films. Samples with 0% and 10% wt of glycerol were brittle, difficult to handle, and required care in peeling from the casting surface. In contrast, films with 20% and 30% wt glycerol were flexible and easy to peel and manipulate [20]. 

Water sorption isotherms revealed that films obtained from water kefir grains were less hydrophilic than other materials based on biopolymers. Water sorption isotherms and thermogravimetric analysis showed that the increase in the amount of glycerol in the film produced a global increase on film hydration and a greater mobility to water molecules. This behavior was manifested in the increase in water vapor permeability of water kefir films with the amount of glycerol [20]. Barrier properties against water vapor of water kefir films resulted comparable with other biopolymeric materials [20,55,94].

**Figure 4:**
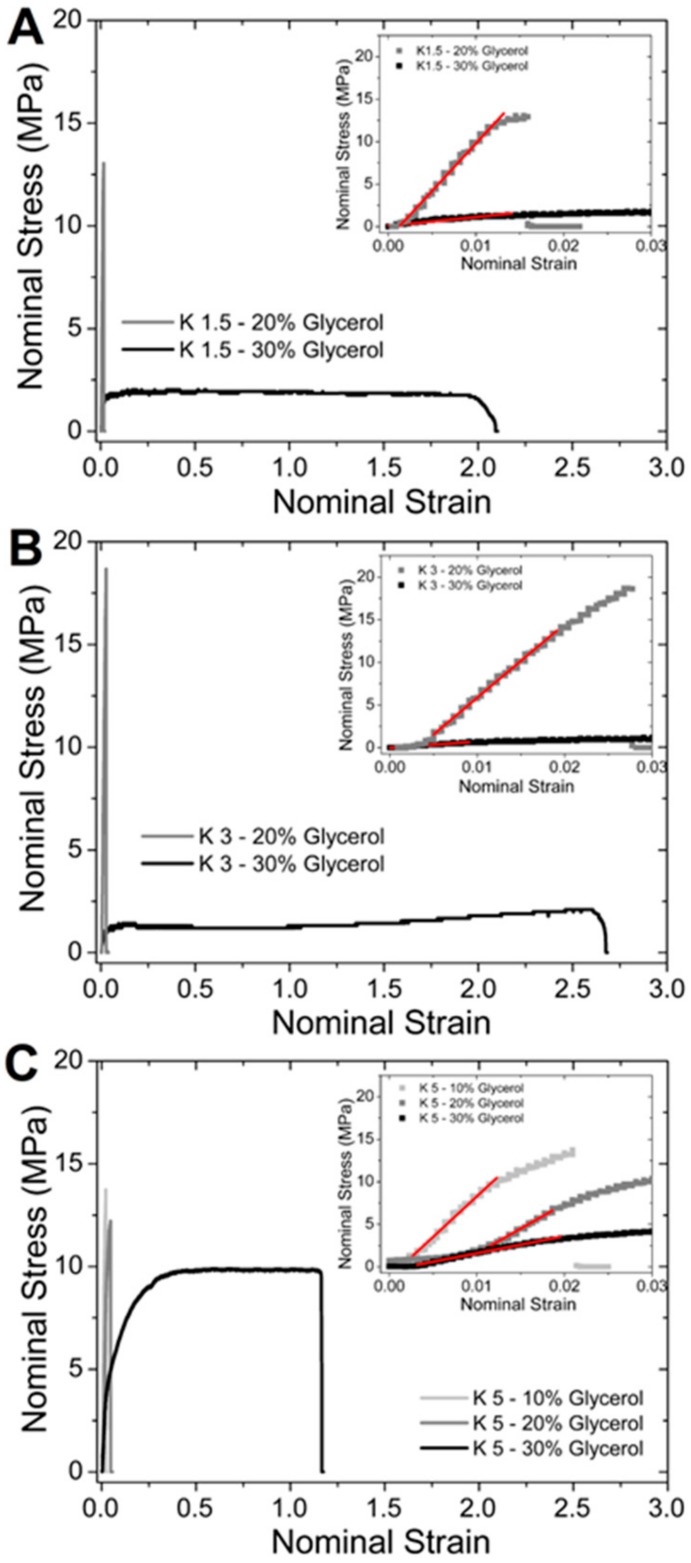
Stress–strain curves of water kefir films with different content of glycerol. Formulations K1.5, K3, and K5 were obtained from film-forming dispersion of (**A**) 1.5, (**B**) 3, and (**C**) 5 wt% dry matter of water kefir grains, respectively.

The presence of plasticizer had a great impact on thermal properties decreasing T_g_ of the dried films from 131 ± 2 °C for plasticized samples to 47 ± 2 °C for samples plasticized with 35% wt glycerol [20]. These values of T_g_ were noticeably higher than T_g_ measured for kefiran films, where the T_g_ was −15 °C for unplasticized and −22 °C at the same level of plasticizer [83]. This difference suggested that water kefir films presented more vitreous characteristics than kefiran films. Furthermore, the plasticizer affected the mechanical properties of water kefir films remarkably, as can be seen in Figure 4 and Table 2. In addition to this, mechanical properties were affected by the initial concentration of the polymer in the film-forming dispersions. The initial concentration of film-forming dispersions affects inter-polymer interactions during the casting process (solvent evaporation) affecting the final structural arrangement of the matrix film. In Table 2 and Figure 4, mechanical properties of water kefir films prepared with different concentration of dry mass, 1.5% (K1.5), 3% (K3) and 5% (K5), are shown. It can be observed in Table 2 that the addition of 30 wt% of glycerol improved significantly the elasticity of films, especially in the formulation K3 (3% dry base) that achieved a deformation at break of 275 ± 15%. This behavior clearly exemplifies that the plasticizer works at the molecular level which produced a decrease in the cohesive forces between polymer chains, allowing greater mobility and enhancing film flexibility. The deformation at break observed in water kefir film formulations K1.5 and K3 with 30 wt% of glycerol was clearly higher than those values obtained for other biodegradable films with significant properties of elongation at the same plasticizer concentration [20]. Moreover, this value was higher than the values registered for films based on kefiran plasticized with 35% wt of glycerol (~170%) [82,87] and high-density polyethylene (150 ± 8%) [95].

These studies showed the potential of the entire water kefir grains to be used in the development of new biodegradable materials. These materials should be considered as an alternative option to traditional sources of biopolymers, without further purification.

### 3.5. Applications of Kefiran and Dextran-Based Materials

**Figure 5:**
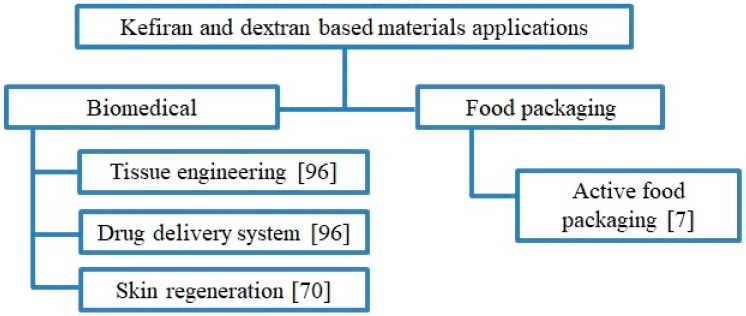
Applications of kefiran and dextran biobased materials.

Different applications of kefir granules are described in the bibliography, and they are mainly focused on two main fields: biomedical [70,96] and food packaging technologies [7]. Some examples are described in Figure 5.

## 4. Cellulose from Microbial Sources: Bacterial Cellulose

During recent years, material scientists and engineers have studied new materials based on biomass sources to develop sustainable materials for technological applications. Different polysaccharides are suitable biopolymers due to their film-forming ability; cellulose is the most common choice for natural polymeric materials because it is the most abundant polysaccharide in nature. It is isolated mainly from plants and must be purified to convert it into different high-value products with low environmental impact [20,97]. This purification process to extract pure cellulose from vegetable sources by removing impurities as lignin and hemicellulose is highly energy demanding. It induces polymer degradation decreasing yields, and the environmental impact of the process’s residues needs to be considered. In contrast, cellulose obtained from microbial sources has several advantages over plant cellulose, mainly due to its extraordinary purity and nanometer size of the fibers that constitute the cellulosic network [98]. 

A.J. Brown [99] reported the first study on microbial cellulose during his investigation of the vinegar fermentation process in which a systematic formation of gelatinous strong floating membrane on culture broth was observed that he called “vinegar plant”. The “vinegar plant” exhibited the characteristic reactions for cellulose with potassium hydroxide (insolubility), sulphuric acid in iodine (no color), and zinc chloride with iodine (blue color) and also demonstrated that the product obtained from acid hydrolysis reduced Fehling’s solution, concluding the production of reducing sugars as dextrose, being the main monomer of the material. In subsequent years, while vinegar fermentation occurs, the cellulosic material constituted a process by-product of the French method to produce wine vinegar, also known as Orleans process, but it was not utilized [100]. Similarly, a fermented tea beverage gained in popularity for its detoxifying and healing properties. This beverage has been denominated Kombucha tea. Up to now, it has been prepared by the infusion of black tea added with sucrose and inoculated with a previous culture. After incubation during 12 to 15 days at 20–26 °C, a “tea fungus” is formed, which is like a gelatin that covers the surface of a sparkling broth consumed as a probiotic beverage. The “tea fungus” is not a microorganism. It is a by-product composed of cellulose and some organic acids that are typical for acetic acid fermentation. This side-stream could serve as an inoculum for new cultures, but it is usually discarded [101]. The cellulose has been identified and characterized using different methodologies as FTIR, thermogravimetric analysis (TGA), X-ray diffraction, dynamic light scattering, and SEM images [102]. Therefore, these studies were the starting points to analyze and characterize microbial cellulose, to explore different technological applications, and nowadays, to develop valuable nanomaterials. 

The reasons for considering microbial cellulose as an attractive biobased material are the conformational structure and enhanced properties compared to plant cellulose. Its chemical purity, nanoscale fibrous network, high water-holding capacity, hydrophilicity, high degree of polymerization and crystallinity index, good chemical stability, transparency, biocompatibility, renewability, biodegradability, and superior mechanical strength are the extraordinary features that offer several advantages when it is used as a native polymer or in composite materials [98,102,103,104].

The main advantage of bacterial cellulose is its purity, greater than 99%, because it does not contain lignin, pectin, hemicellulose, and organic residues associated with plant cell wall; cellulose of plants is obtained with a maximum purity of 80%. Bacterial cellulose features have attractive attributes, such as high nanometer size of the fiber (plant-based cellulose is in the micrometer range), crystallinity (80%–90%) compared to plant cellulose, which has 40%–80% of crystallinity. The degree of polymerization of bacterial cellulose varied from 4000 to 16,000 of anhydroglucose units, while the vegetable source has a degree of polymerization that does not exceed 8000 and in most sources is 1000 units. Regarding mechanical properties, bacterial cellulose has a tensile strength of 1000 MPa versus a maximum of 200 MPa of plant-based cellulose. In the same way, water holding capacity is reported up to 100 times its weight, which means 95% capacity, compared to 35% of plant sources [98,105].

### 4.1. Acetic Acid Bacteria as Cellulose Cell Factory

Different bacteria such as *Acetobacter*, *Gluconacetobacter*, *Komagataeibacter Rhizobium*, *Pseudomonas*, *Erwinia*, and *Agrobacterium* synthesize bacterial cellulose through aqueous culture media. However, bacteria from the acetic acid group (AAB) produce high amounts of extracellular cellulose in the form of pure microfibrils using glucose, fructose, sucrose, and ethanol as the carbon source, while the *Gluconacetobacter* and *Komagataeibacter* genus are the most notable cellulose producers. AAB are mesophilic and strictly aerobic bacteria occurring in sugary, alcoholic, and acidic environments (pH 4 to 6), such as fruits, flowers, and particularly fermented beverages. They participate in partial oxidation of carbohydrates releasing aldehydes, ketones, and organic acids into the surrounding media, through “oxidative fermentations” [103,106,107,108]. 

Bacterial cellulose from AAB has been investigated as native and modified biopolymer in functionalized materials; some aspects of production, such as carbon source, temperature, hydrodynamic conditions, and fermentation time, are important to modulate cellulose production and its morphology. The most recent studies about AAB cellulose-producing species have been to exploit and understand the mechanism to produce cellulose for different uses. In Table 3, some studies regarding the species of *Komagataeibacter* are summarized, which demonstrated to be exceptionally efficient cellulose producers among the AAB group.

The main carbon source to produce bacterial cellulose is glucose; it acts not only as an energy source but also as a cellulose precursor. Table 3 shows that glucose concentration varied from 20 to 100 g/L, resulting in a maximum conversion yield of 0.85 g cellulose/g glucose. Glucose participates in respiratory metabolism, producing gluconic acids and extracellular cellulose as a protective mechanism [103,115]. 

It has also been reported that the addition of ethanol or lactate to the growth media increases the cellulose production in *K. xylinus* since they are linked to the respiratory chain. Lactate and ethanol do not act as cellulose substrates, but they generate extra energy that increases cell density during the early stages of cultivation, resulting in increased cellulose production. During AAB cultivation to produce cellulose, there must be a balance in the type and concentration of the carbon source to avoid the production of acetic acid, which decreases the pH and avoids the production of the polysaccharide [109,116].

*K. xylinus* is an obligate aerobic microorganism; hence, the air supply system is very important to cell growth and physical structure variations of the produced bacterial cellulose. Agitated systems have been demonstrated to reduce cellulose synthesis, probably due to genetic instability of bacteria under agitated conditions. Further, because of the non-Newtonian behavior during shaking or mechanical agitation, there is high shear stress that affects cellulose synthesis and exportation to the external environment. The cellulose produced by agitated culture results in macroscopic changes as formation of spherical or aggregated particles of the polysaccharide and produces different microstructure and properties, such as a low degree of polymerization, a low crystallinity index, and inferior mechanical properties [105]. 

On the other hand, static systems with a high area in the air–liquid interface are a convenient method to increase cellulose production in the form of a floating membrane; in this sense, some reports have shown a production up to 19.6 g/L (Table 3). Like the exopolysaccharide acts as cell support by attaching them, the membrane prevents cell dehydration, provides protection to cells from harsh conditions, like as UV radiation, external microorganism contamination, and against antimicrobial compounds by limiting its diffusion and increasing cell density. Cellulose membrane also helps the bacteria to become floatable, via entrapment of carbon dioxide produced in fermentation, allowing the bacteria to reach the air–liquid interface as a mechanism to take oxygen [103,105,110,116].

Gullo et al. [103] depicted the molecular aspects of the cellulose synthase machinery; it includes different subunits working in a concerted way that synthetize and export the cellulose chains to the extracellular space. The cellulose synthase machinery uses activated glucose monomers as a precursor and is regulated by molecules involved in biofilm cycle. 

### 4.2. Kombucha Tea Fermentation Produces Cellulose

Kombucha tea is a probiotic sparkling beverage obtained by the fermentation of an infusion of tea leaves and sucrose with a symbiotic community of bacteria and yeast (SCOBY). During the preparation of this beverage, a floating cellulosic membrane and the broth are formed. The broth is sour and gasified by carbon dioxide produced during fermentation and is considered a wellness beverage. The side stream of this fermentation is the floating membrane that has been identified as bacterial cellulose [101,117,118]. 

Fermentation dynamics in Kombucha tea involve the synergy of the symbiotic community. The metabolic activity of the osmophilic and acid tolerant yeasts (*Zygosaccharomyces*, *Pichia*, *Brettanomyces*, *Saccharomyces*, *Saccharomycodes*, and *Candida*) releases enzymes that hydrolyze sucrose; hence, glucose and fructose are produced that serve as a source of energy for the entire community. Yeasts produce carbon dioxide and ethanol; this activates the AAB metabolism, the main group of bacteria of the SCOBY, to oxidize it to gluconic acids, gain energy, and stimulate bacterial growth to produce cellulose using glucose and fructose as precursor to cellulose synthesis. 

As for the AAB, it has been reported that the symbiotic relationships between these bacteria with yeasts result in higher cellulose production yields, due to the natural and stable conformation of the microbial community or consortium and the synergistic and coordinated metabolism of the different microorganisms allowing an efficient activity of the cellulose synthase machinery [119].

The fermentation of kombucha tea has been widely studied in its components as a probiotic beverage [117,120,121]. However, the study of the cellulose by-product of this beverage has been limited. Table 4 shows a few reports in which the object of study is the cellulose membrane of Kombucha tea fermentation, using a black tea infusion, a previous culture as inoculum, static conditions, and room temperature.

**Figure 6:**
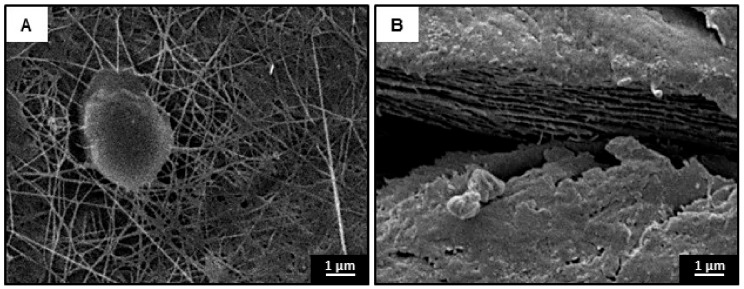
SEM observations of the dried Kombucha cellulose surface at 7000× magnification (**A**) and cross-sections (7000×) (**B**) of cellulose film.

Since there is still a great potential to obtain cellulose from Kombucha tea cultures, our preliminary studies have been conducted in which 10.8 ± 0.5 g/L of cellulose has been obtained from infusion of black tea with 100 g/L sucrose. The macroscopic appearance of the material is a brown-colored gelatinous membrane, consisting of thin layers of the material, at microscopic level, nanometer filaments could be observed. SEM micrographs of the material are shown in Figure 6. 

It is possible to observe in Figure 6A that some filaments with a diameter less than 100 nm and a maximum length up to 30 µm are emerging from the cell, constituting a porous network. Figure 6B shows the cross-section of the formed film, which consists of up to 12 stacked layers of material. The structure observed provides the material with exceptional mechanical properties, giving values of tensile strength 25 ± 2 MPa and a YM of 823 ± 97 MPa [124].

### 4.3. Technological Applications of Microbial Cellulose

**Figure 7:**
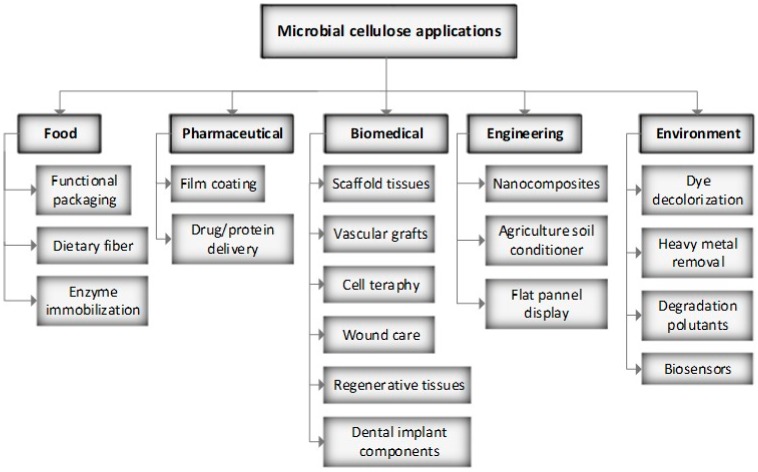
Different application fields of microbial cellulose. Adapted from Hussain et al. [125].

Because of microbial cellulose purity, nanometer fiber network composition, mechanical properties, crystallinity index, water-holding capacity, hydrophilicity, transparency, biocompatibility, and biodegradability, bacterial cellulose is an attractive biopolymer for a number of applications including biotechnological food, biomedical, cosmetics, and engineering fields. It is a promising material for many applications like those shown in Figure 7 that include strength reinforcement of polymeric materials or paper, a thickening agent and food stabilizer, food packaging, biomaterial for manufacturing cosmetics, artificial skin, artificial blood vessels or tissue engineering, and for the preparation of optically transparent films, electric conductors, or magnetic materials [115].

Processed cellulose membranes, because of its mechanical properties, low oxygen transmission rate (barrier property), and its hydrophilic nature, appear to have great application as packaging materials in food packaging, where continuous moisture removal and minimal oxygen transmission properties play a key role [7]. Moreover, cellulose derivatives can be designed for functionally properties as antimicrobial activity. For example, bacterial cellulose films were modified by bovine lactoferrin adsorption. The functionalized films were assessed as edible antimicrobial packaging, for use in direct contact with highly perishable foods, specifically fresh sausage as a model of meat products. *Escherichia coli* and *Staphylococcus aureus* were the model microorganism to inhibit its growth, and bactericidal efficiency was up to 94% [126].

Regarding biomedical applications, cellulose is used as a dressing material due to its high porosity that allows the potential transfer of antibiotics and other medicines into the wound, and at the same time, it serves as an efficient physical barrier against external infection. In addition, bacterial cellulose exhibits properties that are promising for use as artificial skin, scaffold for tissue engineered, vascular grafts, cell, gene and diagnostic therapy. Due to its high purity and net-like morphology similar to human collagen as a biomimetic feature, this facilitates applications. In the same way, cellulose can be used in dental implants, medical pads, artificial bone and cartilage, delivery of drugs, proteins, and hormones [7,127]. 

A research topic was based on new *biocomposites* using bacterial cellulose with chondroitin sulfate, hyaluronic acid, and calcium phosphate for guided tissue regeneration. As bacterial cellulose has –OH groups in its structure, which enable the stabilization of H bond networks and film-forming properties. However, other features are required for biomedical and pharmaceutical applications, such as surface charges; hence, other polysaccharides can be used in composites with cellulose; for example, alginate has a surface negative charge that can be used with cellulose. In similar way, metal nanoparticles have appeared as a new possibility to design cellulose materials. The biological properties of metal nanoparticles of gold, copper, and platinum have been discovered recently and are already being widely used with bacterial cellulose in various biomedical applications [128].

Regarding applications in agriculture, in a dry and semiarid environment, water retention capacity or moisture retention plays a key role in the growth and establishment of crops. Microbial cellulose hydrogel serves as soil conditioners because it is competent to increase water-holding capacity by reducing infiltration rate and improving water conservation of soil, as well as soil cohesion and porosity. The cost-effective, biodegradable, and eco-friendly nature of cellulose makes it an important alternative to synthetic polymers as polyvinyl alcohol [129]. 

Environmental applications are related to cellulose ability to complex with dyes. Particularly bacterial cellulose is excellent biocompatible material that has a less toxic effect compared with a synthetic polymer bind with azo dyes, for example, cellulose grafted with acrylic acid has successfully explored for removal of methylene blue from aqueous solution. Cd and TiO_2_ supported on bacterial cellulose nanofiber were used as photocatalysts for degradation of a model pollutant, methyl orange; cellulose fibers have also been reported for bioadsorption of heavy metals like Pb, Cd, and Ni [130].

### 4.4. Agro-Industrial Residues as Sustainable Substrate for Cellulose Production

Wastes or industrial residual streams have been studied with the aim to produce cellulose, taking advantage to by-products. Therefore, sustainable process to produce a biodegradable polymer, allowed closing the carbon cycle and reducing negative environmental impact. 

The agricultural waste is considered for economic significance, it is a potential source for renewable energy due to environmentally friendly nature, low cost, and sustainability. Husain et al. provide a summary describing culture media to produce bacterial cellulose using different waste products [125]. The corn stalk hydrolysate was used for bacterial cellulose production; it contains glucose, xylose, mannose, furfural, lignin, and acetic acid and 2.86 g/L of cellulose was obtained. Further, hydrolyzed wheat straw having total sugar concentration of 43 g/L was used as a fermentation medium and was obtained 9.7 g/L of cellulose. In the same way, the coconut and pineapple juices, which are discarded as waste by most of the agro-industries, are rich in proteins, carbohydrates, and trace elements. These juices allowed the production of 12.3 g/L of bacterial cellulose. Rice bark, from agricultural residues, was pre-treated with an enzymatic pool and this enzymatic hydrolysate contained a concentration of glucose up to 40 g/L, which was used by an *Acetobacter* strain to produce 2.42 g/L of polysaccharide. To utilize orange peels as a substrate for bacterial cellulose production, it was pre-treated with cellulase and pectinase to increase the concentration of fermentable sugars (60–80 g/L). The results demonstrated cellulose production enhanced by six times compared to formulated standard (HS) medium. 

The brewery and beverages industries produce large volumes of by-products. The use of industrial by-products will not only solve the major waste disposal problem of these industries, but it can also help in the upscale production and commercialization of cellulose bacterial based products. As an example, brewery molasses yielded up to 3 g/L of cellulose. In addition, wine production is one of the most important agricultural activities throughout the world. The wine industry consumes a considerable amount of resources and produces a large amount of waste water and organic wastes characterized by high contents of biodegradable compounds and suspended solids. Among them, grape pomace is the most abundant residue left after juice extraction and does not require difficult or expensive pre-treatments. Grapes provide the fermentable sugars D-glucose and D-fructose that can be used as renewable carbon and energy sources as well as cellulose precursor supplemented with corn steep liquor as the main nitrogen source, the mixtures allowed obtaining 7.2 g/L of bacterial cellulose [130]. Another eco-friendly green method for biosynthesis of bacterial cellulose was proposed by Yong-He Han et al. [131], using a low-cost carbon source from the shell extract of *Sapindus mukorossi*, which after 7 days of *Komagataeibacter xylinus* incubation produced 1.31 g/L of cellulose.

## 5. Conclusions

All through this review novel sources of biopolymers to develop biodegradable materials were described. Microbial biomass and its derivatives demonstrated high potential to perform new materials, which can be formulated and modified to reach the desired properties or enhanced them. The importance of this topic is that, in nature, there is a huge spectrum of biopolymer sources able to perform new materials not fully exploited today. These non-traditional sources of biopolymers could contribute to scientists and technologists to offer better services and goods and reduce our impact on nature. The challenge for researchers and the industry is to take advantage of their potential by proposing new processes and formulations, in order to adapt the properties of these new biobased materials for any specific application. 

## Figures and Tables

**Table 1:** Different applications of fungal biomass.

Fungal Biomass	Applications	Details	References
**Yeast Biomass**	Encapsulation of bioactive compounds	Encapsulation of volatile molecules as flavors to guarantee permanence during the industrial process and probiotics in order to optimize its viability.	[59,61]
	Drug delivery system	Yeast microcapsule used in the delivery of charged nanoparticles: quantum dots, gallium nanoparticles, and various fluorescent nanoparticles	[66]
	Wound dressing sheets	Yeast β-glucan for development of dressing sheets for wound healing (tested on mouse skin)	[67]
**Mycelium Biomass**	Automotive applications	Replace foams in bumpers, doors, roofs, engine bays, etc.	[28,68]
	Packaging materials	Packaging materials that are environmentally responsible, food wrapping. Alternative to traditional polystyrene and polyurethane	[69]
	Electrical circuit boards	Sheet of mycelium containing metal salts of CuSO_4_, CuCl_2_, or Al_2_O_2_	[28]
	Construction and Building Materials	Insulation, structural insulating panels (SIPs), acoustical tiles	[28,68]
	Textile and paper industry	Potential use of fungal pulp in the production of textiles	[28]
	Home and garden	Containers, garden planters, wine shippers, candle holders	[68]

**Table 2:** Tensile parameters YM, TS, and *e*% obtained for water kefir films formulations K1.5, K3, and K5. Values are an average from ten replications experiments for each specimen (*p* < 0.05).

Formulation	% wt Glycerol	YM [MPa]	TS [MPa]	*e*_%_
K1.5	20	1118 ± 27	13 ± 1	1.6 ± 0.2
K1.5	30	103 ± 8	2.0 ± 0.4	215 ± 14
K3	20	900 ± 15	13 ± 1	2.5 ± 0.2
K3	30	54 ± 6	1.9 ± 0.3	275 ± 15
K5	10	956 ± 21	14 ± 1	2.0 ± 0.2
K5	20	585 ± 16	12 ± 1	4.8 ± 0.4
K5	30	201 ± 11	10 ± 1	71 ± 11

**Table 3:** *Komagataeibacter* species cultures to produce cellulose, using glucose as main carbon source. The conversion yield is expressed as gram of cellulose per gram of glucose consumed.

*Komagataeibacter* Species	Glucose	Temperature	Culture Time	Cellulose	Yield	Productivity	Reference
	(g/L)	(°C)	(days)	(g/L)	(g/g)	(g/L·d)	
*K. xylinus*	100	28	10	19.6	0.61	1.96	[109]
*K. hansenii*	20	30	3	6.5	0.33	2.17	[110]
*K. xylinus*	50	30	7	1.8	ND	0.26	[111]
*K. rhaeticus*	20	30	5	4.4	0.25	0.88	[112]
*K. medellinensis*	20	ND	8	2.8	0.20	0.35	[113]
*K. xylinus*	20	28	7	17.0	0.85	2.43	[114]

**Table 4:** Kombucha tea preparation for cellulose production.

Black Tea	Carbon Source	Cellulose	Reference
(g/L)	(Type and Concentration)	(g/L)	
5	High fructose corn syrup 12%	3.5	[102]
5	Sucrose 100 g/L	6.4	[122]
6	Sucrose 90 g/L	6.2	[123]
